# Sturgeon (*Acipenser*)-Derived Chondroitin Sulfate Suppresses Human Colon Cancer HCT-116 Both In Vitro and In Vivo by Inhibiting Proliferation and Inducing Apoptosis

**DOI:** 10.3390/nu12041130

**Published:** 2020-04-17

**Authors:** Ruiyun Wu, Nan Shang, Meng Gui, Jian Yin, Pinglan Li

**Affiliations:** 1 Beijing Advanced Innovation Center for Food Nutrition and Human Health, Key Laboratory of Functional Dairy, College of Food Science and Nutritional Engineering, China Agricultural University, Beijing 10083, China; wry0814@cau.edu.cn (R.W.); yinjian398144775@163.com (J.Y.); 2 Department of Agricultural, Food and Nutritional Science, University of Alberta, Edmonton, AB T6G 2P5, Canada; nshang@ualberta.ca; 3 Beijing Fisheries Research Institute, Beijing 10083, China; guimeng172@126.com

**Keywords:** sturgeon, chondroitin sulfate, HCT-116 cell, tumor xenograft, apoptosis

## Abstract

Chondroitin sulfate (CS), mainly present in the cartilage and bone of animals, is known as a potential food-derived bioactive that has several biological functions, such as anti-arthritic and anti-inflammatory activity. Sturgeon (*Acipenser*), an important fishery resource in China, contains an abundance of CS in their cartilage. In our previous study, we have extracted and purified CS from sturgeon cartilage. Herein, we further investigate the health benefits of sturgeon-derived chondroitin sulfate (SCS), especially for colorectal cancer treatment. The in vitro study indicated that SCS could inhibit the proliferation of the human colon cancer cell line HCT-116 in a dose-dependent manner, which was associated with cell cycle arrest. In addition, SCS also led to extensive cellular apoptosis in colon cancer cell HCT-116 cells. Meanwhile, an in vivo study showed that SCS treatment significantly inhibited the tumor development of xenograft HCT-116 in mice via proliferation suppression and apoptosis induction. Further, a mechanistic study demonstrated that the apoptosis induction was mainly due to the activation of the *Bcl-2* family-associated mitochondrial pathway. Overall, our results provided a basis for SCS as a promising agent against colon cancer.

## 1. Introduction

Colorectal cancer (CRC) is the third most commonly diagnosed cancer in the world and could lead to more than 100,000 death annually [1]. The number of cases will rise over the following two decades as a consequence of the aging and growth of populations all over the world. In past decades, substantial progress has been made in the development of treatment options, which have radically changed the median overall survival of CRC patients. The mainstay of CRC treatment remains the use of cytotoxic agents, as well as irinotecan or oxaliplatin, which results in an average survival of 18 months when combined with 5-fluorouracil (5-FU) and leucovorin or capecitabine [2]. Although available treatment options reduce the incidence and mortality of CRC, they are limited in treatment efficacy as well as associated with serious long-term side effects [3]. Thus, it is important to develop new approaches for CRC inhibition and treatment.

CRC has been considered as one of the clearest markers of the cancer transition, replacing infection-related cancers in countries undergoing rapid societal and economic changes together with other cancers predominantly linked to western lifestyles, which are already frequently found in high-income countries [4]. Epidemiology shows that the rapid increase in both CRC incidence and mortality are now observed in many medium-to-high human development index (HDI) countries, particularly in Eastern Europe, Asia, and South America, mainly due to the assimilation of “the Western dietary pattern”, containing large amount of red meat, which emphasizes the impact of diet [5]. In contrast, the incidence and mortality rates have been stabilizing or declining in a number of highest-indexed HDI countries, such as the USA, Australia, New Zealand, and several western European counties [6]. The reasons for the declining trends in these countries partially reflect increased early detection and prevention through polypectomy, improvements in perioperative care, chemotherapy and radiotherapy, as well as the dietary intervention and supplementation of nutraceuticals [7]. There are data suggestive of a causal relationship between high intake of n-3 long-chain polyunsaturated fatty acids (LC-PUFA) and reduced risk of CRC, indicating that fish intake probably has a CRC protective effect [8]. In addition, the role of nutritional supplementation and bioactive compounds are also considered as an approach for CRC prevention and treatment. Studies claimed the positive effects of calcium supplementation, vitamin D, antioxidant vitamins (β-carotene, vitamins C and E) in reducing the risk of colorectal adenomas.

Chondroitin sulfate (CS) belongs to the group of glycosaminoglycan and is the major constituent of cartilage [9]. Proteoglycans containing CS polysaccharide chains are ubiquitous, but CS is located mainly in cartilage and the calcification site of the bone [10]. CS has been widely used for medical and nutraceutical purposes, such as osteoarthritis treatment, cardiovascular disease, tissue regeneration and engineering, due to its role in regulating cellular activity and maintaining tissue structural integrity [10,11]. Recently, the relationship between chondroitin sulfate and cancer prevention has been studied and it has been reported that CS plays a major role in breast cancer metastasis [12] and breast cancer cell invasion by suppressing activation of the N-cadherin/β-catenin pathway [13]. Chondroitin sulfate is also be considered a molecular portal that preferentially mediates the apoptotic killing of tumor cells [14]. Furthermore, some studies have reported anti-inflammatory effects of chondroitin sulfate, which may be a possible role of chondroitin sulfate in cancer prevention [15,16].

Although several studies have investigated the general anti-cancer effects of chondroitin sulfate, only little is known about the ability of chondroitin sulfate to inhibit CRC. Thus, in this study, we investigate the effects of a hybrid sturgeon (*Acipenser baeri × Acipenser schrenckii*)-derived chondroitin sulfate (SCS) on CRC prevention and treatment both in vitro and *in vivo.* The human colon cancer cell line HCT-116 was used to evaluate the in vitro inhibition effect of SCS on regulating cell proliferation and apoptosis. Meanwhile, HCT-116 cells were also used to induce xenograft tumors in mice and to validate the in vivo anti-cancer effect of SCS as well as to study the underlying molecular mechanisms.

## 2. Materials and Methods

### 2.1. Material and Reagents

Artificial breed hybrid sturgeon (*Acipenser baeri × Acipenser schrenckii*) was obtained from the Sturgeon Farm of Beijing Fisheries Research Institute (Fangshan, Beijing, China) and immediately transported to the laboratory. Skull and spine were separated and collected for chondroitin sulfate extraction. Dulbecco’s modified Eagle medium (DMEM) and phosphate-buffered saline (PBS) were bought from Gibco (Grand Island, NY, USA). Trypsin-EDTA (0.25M), paraffin, hematoxylin, hematoxylin eosin (HE) staining kit were bought from Solebo Technology Co. (Beijing, China). Histonstin-SP IHC kit and mental enhanced DAB substrate kit were bought from Zhongshanjinqiao Biotechnology (Beijing, China). CCK8 cell counting kit was bought from Yisheng Biotechnology (Shanghai, China). Proliferating cell nuclear antigen (PCNA) monoclonal antibody was bought from Abcam (Cambridge County, UK). TdT-mediated dUTP nick-end labeling (TUNEL) kit, caspase 9 activity assay kit, Hoechst 33258, annexin V-FITC apoptosis detection kit, cell cycle and apoptosis analysis kit was purchased from Beyotime Biotechnology (Shanghai, China). Trizol reagent was bought from Invitrogen (Carlsbad, CA, USA). Reverse transcription kit was bought from Booman Biotechnology (Beijing, China). SYBR Fast qPCR kit was bought from Kapa Biosystems (Indianapolis, IN, USA).

### 2.2. Cell Culture

The human colorectal carcinoma HCT-116 (ATCC CRL-247) was purchased from American type culture collection (ATCC) (Manassas, VA, USA). The cells were cultured in DMEM medium with 10% FBS, and 1% penicillin-streptomycin and incubated at 37 °C, 95% O_2_, and 5% CO_2_. After 80%–90% confluency, the cells were subcultured into cell culture plates using 0.25% trypsin-EDTA for the following experiments.

### 2.3. Cell Viability Assay

HCT-116 cells were seeded at a density of 5 × 10^4^ cell/mL into a 96-well plate using the complete growth DMEM medium for overnight. Then, cells were treated with different concentrations (100, 200, 400, 800, 1000 μg/mL) of skull-extracted and spine-extracted chondroitin sulfate, respectively, and 100 μg/mL 5-fluorouracil (5-FU) as a positive control. After 24 h incubation, 10 μL CCK8 was added, and cells were incubated for another 4 h. The cell culture medium was mixed on an orbital shaker for 1 min and measured with a microplate reader (BioTek, Beijing, China) at 450 nm. The inhibition rate (%) was calculated using the following formula:

$$Inhibition\;rate\;\left(\%\right)=\frac{control\;well\;absorbance-absorbance\;of\;the\;experimental\;well}{control\;well\;absorbance-blank\;weel\;absorbance\;}\times 100$$

### 2.4. Cell Cycle Analysis

HCT-116 cells were cultured in 6-well plates with a density of 1 × 10^5^ cell/mL and incubated with different concentrations (100, 200, 400, 800, 1000 μg/mL) of spine-extracted chondroitin sulfate respectively and 100 μg/mL 5-FU as a positive control. After 24 h, the cells were trypsinized and collected by centrifuge for 5 min at 500× *g*. Then, the cell pellets were washed once with ice-cold PBS and collected by centrifugation for the following cell cycle analysis. Briefly, the cell pellets were harvested and fixed with 70% cold ethanol at 4 °C overnight. The fixed cells were washed twice with PBS and re-suspended in 500 μL propidium iodide (PI) staining solution (containing 25 μL PI solution and 10 μL RNase A). After 30 min incubation at 37 °C with protection from light, the cells were processed by the FACScan flow cytometry system (BD Company, NJ, USA) and analyzed by FCS Express software (De Novo Software, CA, USA).

### 2.5. Cell Apoptosis Analysis

HCT-116 cells were cultured in 6-well plates with a density of 1 × 10^5^ cell/mL and incubated with different concentrations (100, 200, 400, 800, 1000 μg/mL) of spine-extracted chondroitin sulfate respectively and 100 μg/mL 5-FU as a positive control. After 24 h, the cells were trypsinized and harvested by centrifuge for 5 min at 1000× *g* and re-suspended with 195 μL annexin V–FITC binding buffer, 5 μL annexin V–FITC and 10 μL PI solution. The mixture was incubated at room temperature for 10–20 min with protection against light. The cells were processed by FACScan flow cytometry system (BD Company, NJ, USA) and analyzed by FCS Express software (De Novo Software, CA, USA).

### 2.6. Animal Experiment

A total of 60 4-week-old male BALB/c nude mice (Charles River, Beijing, China) were fed chow and water *ad libitum*. The mice were acclimated to the housing conditions (temperature of 22~26 °C under a 12-h light/dark cycle) for 7 days before the development of the CRC model. All experimental procedures were designed in accordance with the U.S. NIH Guidelines for the Care and Use of Laboratory Animals and the study protocol (SYXK (Jing) 2015-0046) approved by the Department of Laboratory Animal Science Ethics Committee of Peking University (Beijing, China).

The experimental CRC model was established via HCT-116 xenograft. Briefly, HCT-116 cells were harvested from cell culture flask and suspended to the concentration of 1 × 10^8^ cell/mL. For each of the mice, 100 μL cell suspension or saline was injected subcutaneously into the right axillary region, except the control group was injected into normal saline. When the subcutaneous xenograft tumors reached 3 mm in diameter, tumor volumes were calculated according to the following formula: Tumor volume (mm^3^) = (length × width^2^)/2. The mice were randomly divided into 5 groups with different treatment for 4 weeks. (1) Untreated group (Untr): intragastric administration of saline; (2) 5-FU group: intragastric administration of 5-FU; (3) High dose (400 μg/g): intragastric administration of 400 μg/g body weight/day of spine-extracted chondroitin sulfate; (4) Medium dose (200 μg/g): intragastric administration of 200 μg/g body weight/day of spine-extracted chondroitin sulfate; (5) Low dose (100 μg/g): intragastric administration of 100 μg/g body weight/day of spine-extracted chondroitin sulfate. The body weight, food intake, and tumor size were measured every week, and the mice’s survival was monitored daily during the experimental period. At the end of the experiment, all animals were sacrificed, and tissues were collected for the following experiments. All the tumors were bisected, one part was fixed in 10% formalin and paraffin-embedded for immunohistochemical (IHC) staining, and the other was snap-frozen and stored in liquid nitrogen for RNA extraction.

### 2.7. Immunohistochemical (IHC) Analysis

Serial sections of 5 μm in thickness were cut from the formalin-fixed and paraffin-embedded xenograft tumor samples for IHC staining. The slides were washed three times with PBS and incubated in 5% normal goat serum to block nonspecific background staining. Sections were then incubated with rabbit anti-PCNA (proliferating cell nuclear antigen) antibody at 4 °C overnight. Then, the sections were washed 3 times before incubated with goat anti-rabbit IgG secondary antibody for 30 min at room temperature, whereas hematoxylin was used for nuclear counterstaining. The sections were examined by microscopy using AxioVer A1 (Carl Zeiss Microscopy, NY, USA) at ×100 magnification, and the images were analyzed using Image Pro Plus v.6.0 Software (Media Cybernetics, MD, USA).

### 2.8. TUNEL Staining Analysis

Serial sections of 5 μm in thickness were cut from paraffin-embedded tissue were deparaffinized with xylene twice for 10 min, hydrated using an ethanol gradient (100% ethanol for 5 min, 90% ethanol for 2 min, 70% ethanol for 2 min, and ddH_2_O for 2 min), incubated with proteinase K for 10 min at room temperature, and subsequently blocked at 3% H_2_O_2_ in PBS for 20 min at room temperature. Then TUNEL staining analysis was performed according to the manufacturer’s protocol. Briefly, samples were incubated with 50 μL biotin labeling solution (45 μL Biotin-dUTP with 5 μL TdT) for 60 min at 37 °C with protection against light. Then, samples were incubated with 50 μL streptavidin-HRP solution for 30 min at room temperature and then DAB solution for 15 min at room temperature. Subsequent to washing with PBS, the sections were counterstained with hematoxylin. Apoptotic cells were examined by microscopy using AxioVer A1 (Carl Zeiss Microscopy, NY, USA) at ×100 magnification, and the images were analyzed using Image Pro Plus v.6.0 Software (Media Cybernetics, MD, USA).

### 2.9. Total RNA Extraction and Quantitative Real-Time PCR (qPCR) Analysis

Total RNA was extracted from the xenograft tumor with TRIzol reagent according to the manufacturer’s protocol. The RNA quality was detected by A_260_/A_280_ and gel electrophoresis. Equal amounts of total RNA were used to synthesize cDNA with the PrimeScript 1st strand cDNA synthesis kit (Takara, Japan). Quantitative real-time PCR was performed in triplicate using SYBR Fast qPCR kit (Kapa Biosystems, MA, USA) and 7500 Fast Real-time PCR system (Applied Biosystems, CA, USA). Gene-specific primers were listed in Table 1. The fold change of the treatment group versus the control group for each target gene was calculated using the 2^−ΔΔCt^ method and was evaluated as the effect of treatment on relative gene expression. The expression was normalized against the expression of the housekeeping gene β-actin.

### 2.10. Statistical Analysis

All data are presented as mean ± SEM (standard error of mean). Data were analyzed using one-way analysis of variance (ANOVA) with Dunnett’s posthoc test for comparisons to control. The PRISM 6 statistical software (GraphPad Software, San Diego, CA) was used for the analyses. *p* < 0.05 was considered significant.

## 3. Results

### 3.1. Sturgeon-Derived Chondroitin Sulfate (SCS) Inhibits the Proliferation of HCT-116

**Figure 1:**
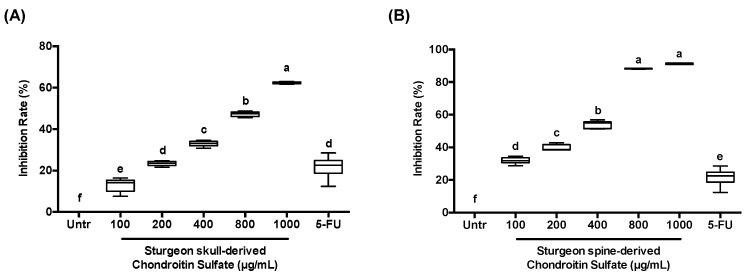
Effect of chondroitin sulfate (SCS) on cell proliferation in colon cancer HCT-116 cells. Colon cancer HCT-116 cells were treated with different concentration (100, 200, 400, 800, and 1000 μg/mL) of SCS and 5-fluorouracil (5-FU; 100 μg/mL) for 24 h. Cell viability was measured by CCK8 cell counting assay and the results were expressed as inhibition rate (%) compared to untreated cells. (**A**) SCS extracted from sturgeon skull, (**B**) SCS extracted from sturgeon spine. All results are expressed as mean ± SEM (*n* = 3). Different superscript letters for each column indicate significant differences (*p* < 0.05) as compared to every other group.

To investigate the anti-proliferative effect of sturgeon-derived chondroitin sulfate (SCS), we executed cell counting assay (CCK-8 kit) against CRC cell HCT-116. The chondroitin sulfate extracted from both sturgeon skull and spine resulted in a significant inhibition of cell growth in a dose-dependent manner (Figure 1). The spine-derived chondroitin sulfate (Figure 1B) showed better inhibition effect with IC_50 (24 h)_ of 237.64 μg/mL, compared to the skull-derived chondroitin sulfate (Figure 1A) with IC_50 (24 h)_ of 742.47 μg/mL. Thus, spine-derived chondroitin sulfate with concentrations of 100, 200, and 400 μg/mL was selected for the following tests. More inspiringly, the inhibition effect of sturgeon-derived chondroitin sulfate (both from spine and skull) was comparable to 5-FU, a well-used anti-cancer drug; and the effect of sturgeon-derived chondroitin sulfate was more efficient compare to 5-FU.

### 3.2. Sturgeon-Derived Chondroitin Sulfate (SCS) Induces Cell Cycle Arrest of HCT-116

**Figure 2:**
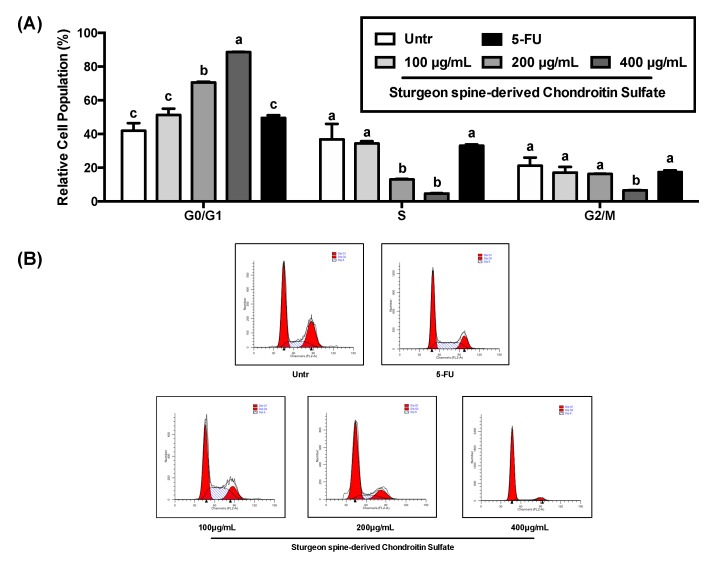
Effect of SCS on cell cycle arrest in colon cancer HCT-116 cells. Colon cancer HCT-116 cells were treated with different concentration (100, 200, and 400 μg/mL) of SCS (spine) and 5-FU (100 μg/mL) for 24 h. The percentages of cells in each phase (G0/G1, S, G2/M) were calculated by cell cycle assay kit and flow cytometer. (**A**) Relative cell population in each phase, (**B**) Representation flow cytometer images. All results are expressed as mean ± SEM (*n* = 3). Different superscript letters for each column indicate significant differences (*p* < 0.05) as compared to every other group.

Cell proliferation is correlated with the regulation of cell cycle progression. Therefore, we determined the effects of SCS on cell cycle arrest on HCT-116 cells. SCS treatment significantly regulated the cell cycle arrest of HCT-116 and therefore affected cell proliferation (Figure 2). SCS treatment increased the accumulation of cell population in the G0/G1 phase from ~40% in untreated cells to ~90% after treated with highest concentration (400 μg/mL) of SCS. At the same time, the percentage of cells in the S phase was significantly decreased from ~40% in untreated cells to ~5% after treatment, and the G2/M phase was significantly decreased from ~20% to ~5%.

### 3.3. Sturgeon-Derived Chondroitin Sulfate (SCS) Induces the Apoptosis of HCT-116

**Figure 3:**
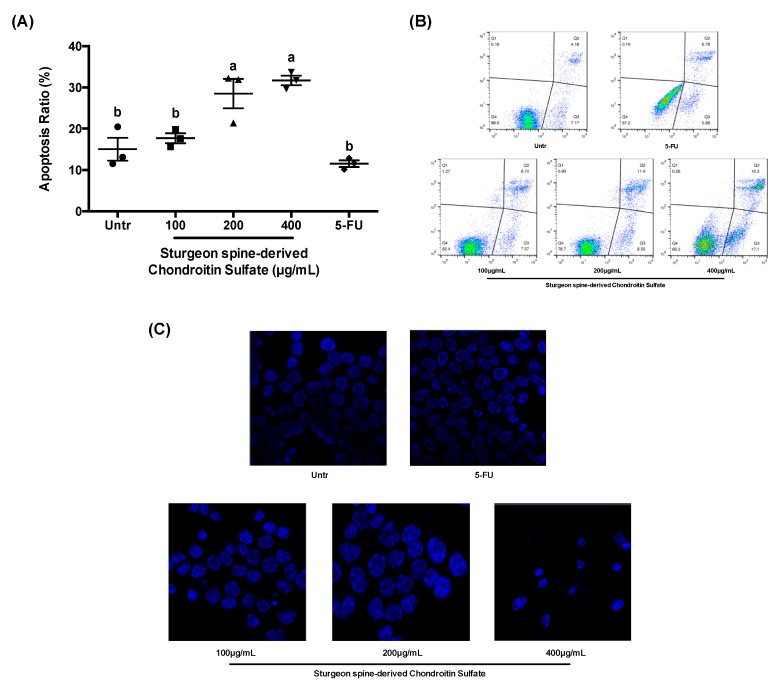
Effect of SCS on cell apoptosis in colon cancer HCT-116 cells. Colon cancer HCT-116 cells were treated with different concentration (100, 200, and 400 μg/mL) of SCS (spine) and 5-FU (100 μg/mL) for 24 h. The apoptosis ratios (%) were calculated by cell apoptosis assay kit and flow cytometer, and the apoptotic bodies were identified by immunofluorescence microscopy. (**A**) Apoptosis ratio, (**B**) representation flow cytometer images, (**C**) representation images of apoptotic bodies. All results are expressed as mean ± SEM (*n* = 3). Different superscript letters for each column indicate significant differences (*p* < 0.05) as compared to every other group.

Apoptosis induction is a broadly accepted approach to control the growth and development of cancer cells. Flow cytometry analysis showed that the HCT-116 cells were induced to apoptotic cell death by SCS (Figure 3A,B). The ratio of apoptotic cells was significantly increased up to ~30% in both 200 and 400 μg/mL SCS treatment, compared to untreated cells. Moreover, the immunofluorescence microscopy further confirmed the stimulatory effect of SCS on cell apoptosis (Figure 3C). Compared with the untreated group, the HCT-116 cells exhibited a marked increase in apoptosis following treatment with SCS, exhibiting nuclear condensation, DNA fragmentation, and the formation of apoptotic bodies.

### 3.4. Sturgeon-Derived Chondroitin Sulfate (SCS) Suppresses the Growth of HCT-116 Tumor Xenograft In Vivo

**Figure 4:**
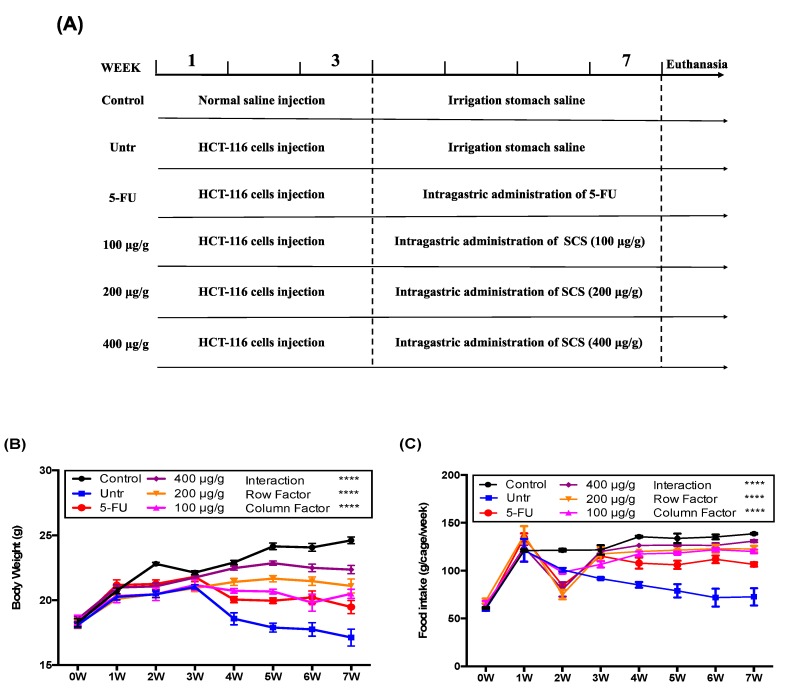
Effect of SCS on body weight and food intake in HCT-116 xenograft tumor mice model. Colon cancer HCT-116 cells were injected into BALB/c nude mice to develop the CRC model, while the control group was injected with normal saline. After 3 weeks, mice received an intragastric administration of different doses (100, 200, and 400 μg/g/day) of SCS (spine) and 5-FU (100 μg/g/day) as a positive control for 4 weeks. (**A**) Diagram of the experimental design, (**B**) body weight, (**C**) food intake. All results are expressed as mean ± SEM (*n* = 10). **** indicates *p* < 0.0001 as compared to every other group.

**Figure 5:**
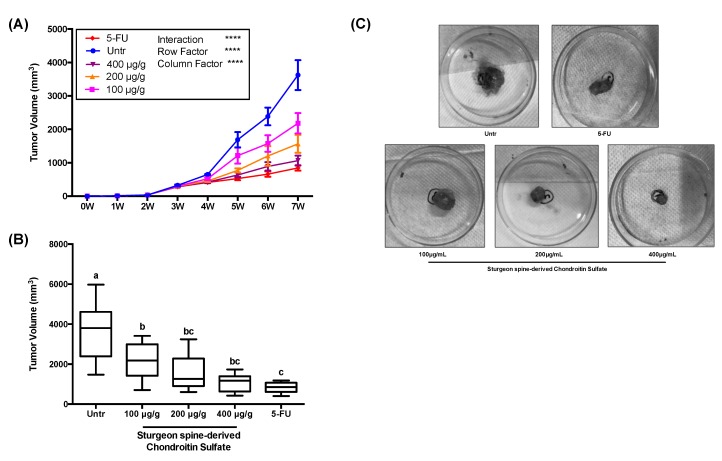
Effect of SCS on tumor volume in HCT-116 xenograft tumor mice model. During the 7-week experiment, the tumor volume of each animal was measured every week and the final tumor volume was measured after sacrifice. (**A**) Tumor volume in each time point, **** indicates *p* < 0.0001 as compared to every other group, (**B**) final tumor volume, (**C**) representation images of tumors from each group. All results are expressed as mean ± SEM (*n* = 10). Different superscript letters for each column indicate significant differences (*p* < 0.05) as compared to every other group.

To study the inhibition effect of SCS on tumor growth *in vivo*, a tumor xenograft model was established in the present study. Based on the preliminary tests (data not shown), we selected 100, 200, and 400 μg/g body weight/day as SCS treatment doses and 7 weeks as the duration of this experiment (Figure 4A). All mice had increased body weight by the end of the experiment, except the untreated group, while orally administration of SCS preserved the bodyweight loss caused by tumor growth (Figure 4B). The administration of SCS also prevented the decrease in food intake caused by tumor growth (Figure 4C). After 3 weeks of HCT-116 cell injection, the tumor xenograft model was successfully developed in all injected animals, in all of which the tumor volume reached ≥30 mm^3^. Then treatment groups were administrated with different doses of SCS and 5-FU as a positive control. After 2 weeks of treatment, the average tumor volume in the untreated group began to differ from that of other treatment groups (Figure 5A). At the end 7 weeks before the sacrifice, the average tumor volume in the untreated group increased to 3622 ± 445 mm^3^, while the SCS treatment group with low dose and high dose grew to 2180 ± 303 mm^3^ and 1063 ± 147 mm^3^, respectively, compared to the 5-FU group, which had an average tumor volume of 845 ± 81 mm^3^ (Figure 5B,C).

### 3.5. Sturgeon-Derived Chondroitin Sulfate (SCS) Inhibits the HCT-116 Tumor Growth by a Reduction of Proliferation and an Induction of Apoptosis

**Figure 6:**
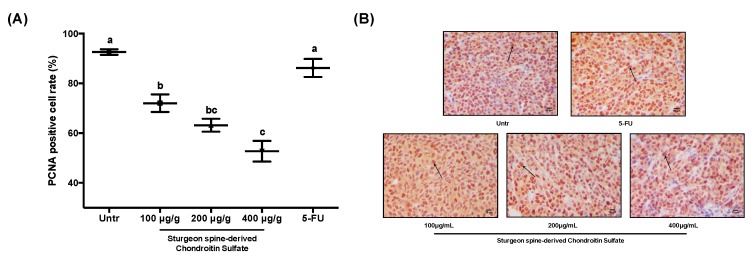
Effect of SCS on cell proliferation in HCT-116 xenograft tumor mice model. Xenograft tumor tissues were collected after sacrifice and sectioned for proliferating cell nuclear antigen (PCNA) staining. (**A**) Percentage of PCNA-positive cells, (**B**) Representation images of PCNA staining. All results are expressed as mean ± SEM (*n* = 10). Different superscript letters for each column indicate significant differences (*p* < 0.05) as compared to every other group.

**Figure 7:**
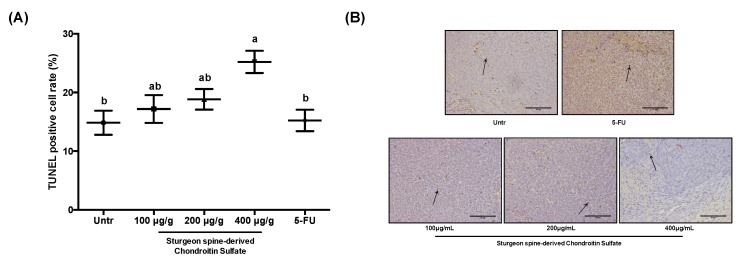
Effect of SCS on cell apoptosis in HCT-116 xenograft tumor mice model. Xenograft tumor tissues were collected after sacrifice and sectioned for TdT-mediated dUTP nick-end labeling (TUNEL) staining. (**A**) Percentage of TUNEL-positive cells, (**B**) Representation images of TUNEL staining. All results are expressed as mean ± SEM (*n* = 10). Different superscript letters for each column indicate significant differences (*p* < 0.05) as compared to every other group.

To further investigate the in vivo effect of SCS on tumor inhibition, we also studied its effects on proliferation and apoptosis in the xenograft tumor. Similar to the in vitro results, the reduction in relative tumor size from our xenograft model of CRC was due to a combination of a reduction in cell proliferation and an induction of apoptosis. PCNA staining revealed that the percentage of PCNA positive cells decreased to 72.04% ± 0.88% and 52.7% ± 1.03% treated with high dose and low dose SCS, respectively, compared to 92.60% ± 0.27% in the untreated group (Figure 6). Meanwhile, the percentage of apoptotic cells in the HCT-116 tumors significantly increased to 25.21% ± 0.47% in the high dose group compared to 14.84% ± 0.51% in the untreated group (Figure 7). More importantly, the effect of SCS on proliferation reduction and apoptosis stimulation was significantly stronger than of 5-FU (Figure 6 and Figure 7).

### 3.6. Sturgeon-Derived Chondroitin Sulfate (SCS) Induces the HCT-116 Tumor Apoptosis via the Mitochondrial Pathway

**Figure 8:**
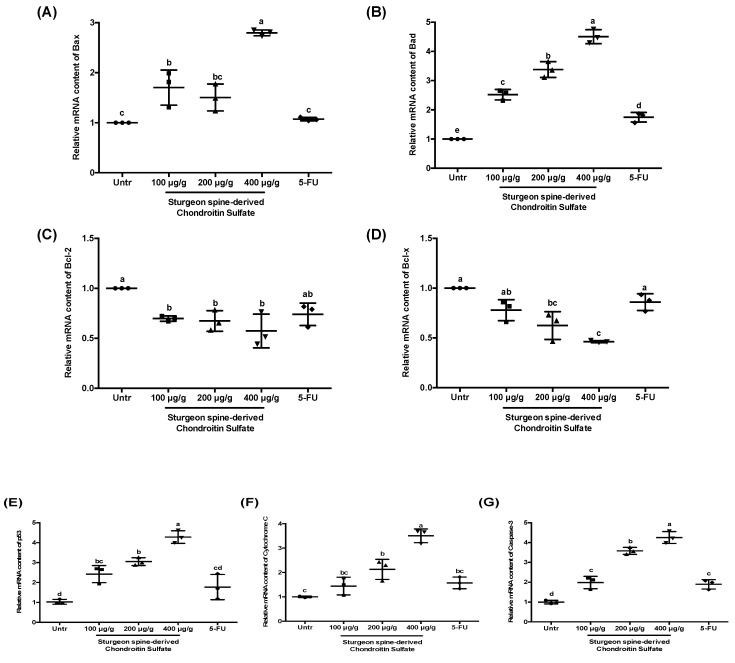
Effect of SCS on regulating mitochondrial pathway in HCT-116 xenograft tumor mice model. Total RNA was extracted from the xenograft tumor and the gene expression of mitochondrial apoptotic makers was measured by qPCR. (**A**) The relative mRNA content of *Bax*, (**B**) *Bad*, (**C**) *Bcl-2*, (**D**) *Bcl-x*, (**E**) *p53*, (**F**) *Cytochrome* c, (**G**) *caspase 3*. All results are expressed as mean ± SEM (*n* = 10). Different superscript letters for each column indicated significant differences (*p* < 0.05) as compared to every other group.

To explore the pattern of SCS-induced apoptosis, the gene expression of key proteins involved in mitochondria-associated apoptosis was measured. As shown in Figure 8, SCS treatment induced an upregulation of pro-apoptotic molecules, *Bax* and *Bad* (Figure 8A,B), with a concomitant constancy or partial reduction in the expression of anti-apoptotic molecules, *Bcl-2* and *Bcl-x* (Figure 8C,D). Meanwhile, we also observed a significant increase of *p53* expression, the master tumor suppressor gene, in SCS treatment groups (Figure 8E). Furthermore, *cytochrome c* and *caspase 3* were also strongly induced by SCS treatment, showing a 3.5-fold (Figure 8F) and 4-fold (Figure 8G) increase in the high dose group, respectively. These results suggest that SCS induces apoptosis via activating the mitochondrial-mediated apoptosis pathway.

## 4. Discussion

**Figure 9:**
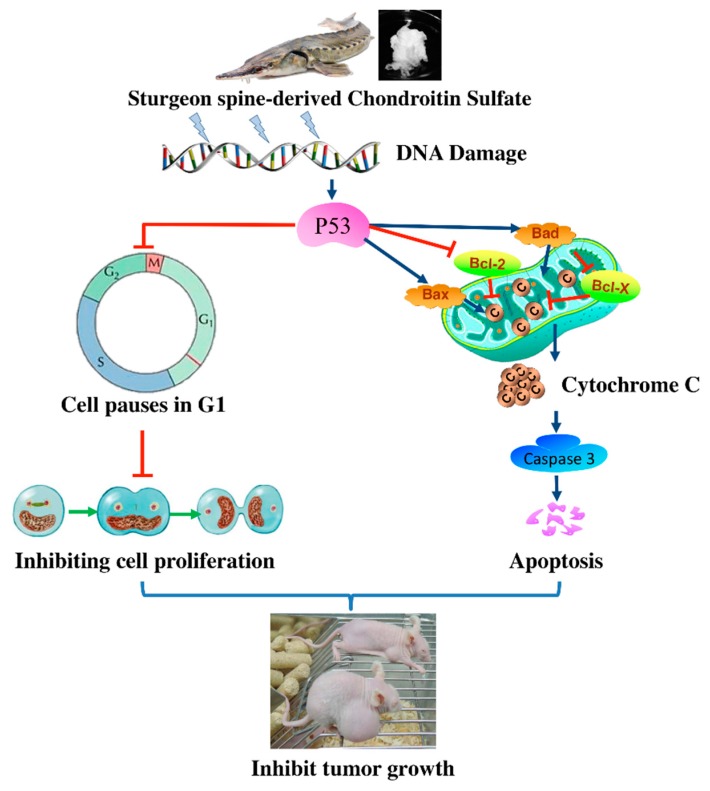
The potential mechanism of SCS-induced inhibition of colorectal cancer progression.

Despite improvements in early detection and treatment methods in recent years, CRC remains the third most common cancer in the Western hemisphere and the fourth leading cause of cancer-associated mortalities worldwide [17,18]. CRC, like numerous other solid tumors, is a heterogeneous disease in which different subtypes may be distinguished by their specific clinical and/or molecular features [19]. The standardized treatment of CRC consists of surgery, chemotherapy, radiation therapy and immunotherapy, which are utilized in accordance with the characters and stages of the cancers [20]. Although there is inspiring progress on CRC treatments in the decades, the therapeutic decisions and chemoprevention strategies remain unsatisfied due to the limited efficacy and associated long-term side effects. Thus, it is important to further extend the understanding of the pathogenesis of CRC, the molecular signaling pathways involved in CRC onset, as well as to develop new strategies and approaches for CRC prevention and treatment. Currently, there are increasing interests in establishing dietary intervention for cancer prevention and treatment due to the investigation and development of anti-cancer bioactive compounds and nutraceuticals. In fact, several conventional chemotherapeutic agents, including taxol, epothilones, and vinca alkaloids, originate from resource of natural products [21]. In this study, we report for the first time that a bioactive compound sturgeon (*Acipenser*)-derived chondroitin sulfate (CSC) shows potential as an anti-cancer candidate drug due to its ability to prevent CRC (Figure 9).

Sturgeon (*Acipenser*) is the largest and most primitive freshwater soft-shelled cartilaginous fish in the world, which can provide abundant cartilage for chondroitin sulfate (CS) extraction. CS has been found with intriguing functions in central nervous system development, wound repair, growth factor signaling, morphogenes and cell division, in addition to osteoarthritis and their conventional structural roles as a member of sulfated glycosaminoglycan (GAG) family [10]. Thus, CS is widely extracted from terrestrial and marine soft tissues and cartilages for the development of nutraceuticals and natural health products [22]. In this study, we found that CSC potently suppressed the proliferation and induced apoptosis of the CRC cell line HCT-116 both in vitro and *in vivo*.

In general, cancer is caused by uncontrolled cell growth and cell division through violating normal cellular function, mainly cell cycle arrest and programmed cell death [23]. The majority of anti-cancer drugs and bioactives are able to inhibit abnormal proliferation and induce cytotoxic activity via cell cycle arrest and stimulation of apoptosis [24]. However, compared to pharmaceuticals, dietary bioactive compounds for cancer prevention are quite safe for normal cell metabolism and effective in accelerating the programmed cell death and interrupting the uncontrolled cell proliferation [25,26]. It is well-known that plant-derived bioactives, especially phenolic compounds, display a strong anti-cancer effect due to their anti-proliferative activity [27,28,29,30,31,32]. Flavonoids have been reported to inhibit cell proliferation and induce apoptosis in human breast cancer [33]. Myricetin, a food-derived bioflavonoid, showed inhibition on cell proliferation of bladder cancer cells by inducing cell arrest at the G2/M phase [34]. Similar cell cycle arrest was also observed in cervical cancer cells following combination treatment with methyl eugenol and cisplatin, while the number of cells in the G0/G1 phase was significantly increased in this case [35]. In our study, CSC significantly inhibited cell viability and proliferation both in vitro and *in vivo*. Cell cycle analysis also revealed that the induction of cell arrest at the G0/G1 phase significantly contributed to CSC-exhibited anti-proliferative activity.

In addition to the suppression of cell proliferation, induction of cell apoptosis is another therapeutic approach for cancer treatment. Especially in CRC treatment, apoptotic pathways have been considered as an essential therapeutic target. In general, cell apoptosis is regulated by three pathways: the intrinsic (or mitochondrial), the extrinsic (or death receptor), and the intrinsic endoplasmic reticulum pathways. In our study, we found that the mitochondrial pathway plays a key role in CSC-induced cell apoptosis [36]. Oral administration of CSC significantly inhibited tumor size and evidently induced the apoptosis of transplanted tumor cells by increasing the TUNEL positive cells. The qPCR analysis reveals that the gene expression levels of *Bax* and *Bad* were increased, while those of *Bcl-2* and *Bcl-x* were decreased, which are all known as mitochondrial-associated apoptotic proteins. All these proteins are members of the *Bcl-2* family, which consists of pro- and anti-apoptotic proteins exerting opposing effects on the mitochondria. *Bcl-2* and Bcl-x are known as anti-apoptotic proteins, which are highly expressed in colorectal carcinomas [37,38]. Moreover, high expression of *Bcl-2* could also lead to resistance to chemotherapeutic drugs and radiation therapy [39]. On the other hand, Bax and Bad are known as pro-apoptotic proteins, which can trigger and promote cell apoptosis but are inactivated in cancer cells [40]. Studies have demonstrated that *Bcl-x* protein plays a significant role in the development of CRC and its metastasis [41]. In a human study, scientists also noticed that Bax-negative patients with CRC show a shorter survival compared to Bax-positive patients [42]. Thus, the effect of CSC on CRC is mainly due to the regulation of *Bcl-2* family member expression and activation of mitochondria apoptosis.

The therapeutic potential of GAGs, such as heparan sulfate/heparin and chondroitin sulfate, for cancer treatment are being actively studied; however, the role and therapeutic application of GAGs seem to be controversial in different studies [43,44]. Recently, Pudelko et al. (2019) reported the dual role of the CS in the development, progression, and metastasis of cancer, and claimed that the effect of CS might significantly depend on its structure, especially the sulfation pattern [44]. CS is formed by the repeating unit (→4GlcAβ1→3GalNAcβ1→), which is then sulfated in different positions of the N-acetyl-galactosamine (GalNAc) unit and/or uronic acid [10]. The dual effect of CS on progression and metastasis of cancer is due to a substantial increase in the CS content and the *6-O-sulfated* and/or unsulfated disaccharide content, which is concomitant with a decrease in the *4-O-sulfation* level. Therefore, the potential effect of CS is significantly depending on the structural properties and structural alternation of CS. In our study, we also observed a difference between skull-extracted SCS and spine-extracted SCS, even though they were extracted from the same species using the same method. Thus, further studies on structure analysis are needed to understand the potential uses of SCS as an anti-cancer agent. Additionally, the sources used for SCS extraction may also have affect its efficacy. In our study, artificially bred hybrid sturgeon (*Acipenser baeri × Acipenser schrenckii*) was used for SCS preparation and the investigation of anti-cancer activity. However, several studies have extracted CS from other sturgeon sources, such as Chinese sturgeon [44], or other fish materials, such as shark cartilage [45], while their bioactivity remains unknown. Besides the source material, the manufacturing processes and final quality of SCS also play extremely important roles in the overall biological, nutraceutical and pharmacological capacities of SCS. The contaminants and other natural biomolecules, such as polysaccharides, proteins, and nucleic acids, may also contribute to the final efficacy of the SCS products [46]. Moreover, as nutraceutical properties may vary with the structure, the oral absorption and delivery method of SCS application is another key issue that can influence the efficacy. Thus, we should consider the use of SCS as a combination therapy with other drugs or with dietary intervention together. Overall, depending on the source origin and the manufacturing processes, variable CS final products for structure, quality, and activity may be produced and show significant differences in their health benefits. Thus, further research on SCS is extensively needed to validate the efficacy of SCS for colon cancer treatment. Meanwhile, another key point for the communalization of SCS is related to the possible side effects for long-term applications. Although CS is considered to be safe, and no serious or fatal adverse events have been reported, several potential side effects have to be kept in mind. Firstly, as the SCS in the current study was extracted from sturgeon, it might lead to allergic reactions in certain individuals. Also, some human and animal studies have suggested that glucosamine and CS can affect glucose metabolism, and insulin resistance has been shown [47]. Therefore, long-term treatment could be risky for diabetic and/or hypertensive patients. Overall, based on the fact that SCS has low and rare adverse effects, it represents a viable option for colon cancer management.

## 5. Conclusions

In this study, we found that sturgeon-derived chondroitin sulfate (SCS) could significantly inhibit the proliferation and induce the apoptosis of colon cancer cell line HCT-116 *in vitro.* Meanwhile, oral administration of SCS resulted in a significant inhibition of xenograft HCT-116 tumor growth in mice via suppressing proliferation and inducing cell apoptosis via the activation of mitochondrial apoptotic cascades. Thus, SCS could be developed as a potential candidate for CRC prevention and treatment.

## Figures and Tables

**Table 1:** Sequences of primers used in real-time qPCR.

Gens	Primer Sequences (5′→3′)
*β-action*	F:CGACCACTTTGTCAAGCTCA
	R:AGGGGTCTACATGGCAACTG
*Bcl-xl*	F:ATGGCAGCAGTAAAGCAAGCGC
	R:TTCTCCTGGTGGCAATGGCG
*Bcl-2*	F:AGATGTCCAGCCAGCTGCACCTGAC
	R:AGATAGGCACCCAGGGTGATGCAAGCT
*Bad*	F:CCTTTAAGAAGGGACTTCCTCGCC
	R:ACTTCCGATGGGACCAAGCCTTCC
*Bax*	F:TCCACCAAGAAGCTGAGCGA
	R:GTCCAGCCCATGATGGTTCT
*Caspase-3*	F:TTTGTTTGTGTGCTTCTGAGCC
	R:ATTCTGTTGCCACCTTTCGG
*Cytochrome C*	F:CCAGGACTGTATGTGGAGCG
	R:CTTGAGGACCAGTGGGCTGT
*p53*	F:TGGCCCCTCCTCAGCATCTTAT
	R:GTTGGGCAGTGCTCGCTTAGTG

## Abbreviations

5-FU: 5-fluorouracil; ANOVA: One-way analysis of variance; CCK-8: Cell Counting Kit-8; CRC: Colorectal cancer; CS: Chondroitin sulfate; DMEM: Dulbecco’s modified eagle medium; FBS: Fetal bovine serum; FITC: Fluorescein isothiocyanate; GAG: Glycosaminoglycan; GalNAc: N-Acetyl-Galactosamine; IHC: Immunohistochemical; LC-PUFA: Long-chain polyunsaturated fatty acids; PBS: Phosphate buffered saline; PCNA: Proliferating Cell Nuclear Antigen; PI: Propidium iodide; qPCR: Quantitative real-time polymerase chain reaction; SCS: Sturgeon (*Acipenser*)-derived chondroitin sulfate; SEM: Standard error of mean; TdT: Terminal deoxynucleotidyl transferase; TUNEL: TdT-mediated dUTP nick-end labeling.
